# Laryngeal Foreign Body Aspiration in Infancy: A Diagnostic Challenge

**DOI:** 10.7759/cureus.60144

**Published:** 2024-05-12

**Authors:** Paula V Guerra, Kelvin Anderson, Sean M Clausen, Michele M Carr

**Affiliations:** 1 Otolaryngology, University at Buffalo Jacobs School of Medicine and Biomedical Sciences, Buffalo, USA

**Keywords:** pediatric foreign body removal, direct laryngoscopy and bronchoscopy, flexible laryngoscopy, glottic foreign body, laryngeal foreign body aspiration

## Abstract

Foreign body aspiration (FBA) is a significant cause of accidental death among children, with laryngeal FBA being relatively rare but potentially fatal due to airway obstruction. This report highlights a case of laryngeal FBA in an 11-month-old child, initially misdiagnosed as viral croup. Otolaryngological evaluation, particularly in the case of laryngeal FBA, may facilitate management.

An 11-month-old male was brought to the emergency department, presenting with inspiratory stridor following a choking episode. A chest radiograph and CT scan of the chest were read as normal. He was suspected of having croup and treated with dexamethasone and racemic nebulized epinephrine, which led to temporary clinical improvement.

The child returned with persistent stridor to the emergency department eight days after his initial visit, prompting an otolaryngological consultation. Flexible laryngoscopy ultimately identified a star-shaped sequin lodged in the glottis. The foreign body was successfully removed via direct laryngoscopy and bronchoscopy (DLB). Following the removal, the patient demonstrated significant improvement and eventually made a full recovery.

This case emphasizes the difficulty in diagnosing laryngeal FBA due to its non-specific symptoms and the limitations of imaging techniques. The importance of a thorough clinical history, physical examination, and proper imaging combined with a high index of suspicion is crucial for early diagnosis and treatment. Additionally, the report discusses the potential for severe complications if diagnosis and treatment are delayed, highlighting the need for awareness and prompt intervention in suspected laryngeal FBA cases.

## Introduction

Foreign body aspiration (FBA) is a significant cause of accidental death among children. Children aged five years and younger are at higher risk of FBA, accounting for 89% of cases [1]. These rates have remained stable over recent years, regardless of extensive public health efforts to warn families about choking hazards [1]. The larynx is an uncommon site of FBA, accounting for approximately 4% of all FBA [2]. Because of the low incidence, laryngeal FBA may be overlooked as a cause of airway obstruction, although there is a risk of mortality and long-term sequelae if not treated promptly.

In this article, we describe a case of an 11-month-old child with a retained glottic foreign body whose symptoms were initially mistaken for viral croup. Otolaryngologist intervention enabled the diagnosis of laryngeal FBA and definitive treatment via foreign body removal. We posit that reporting this case may contribute to a more comprehensive understanding of the clinical manifestations of laryngeal FBA, potentially facilitating timelier interventions by otolaryngologists.

## Case presentation

**Figure 1:**
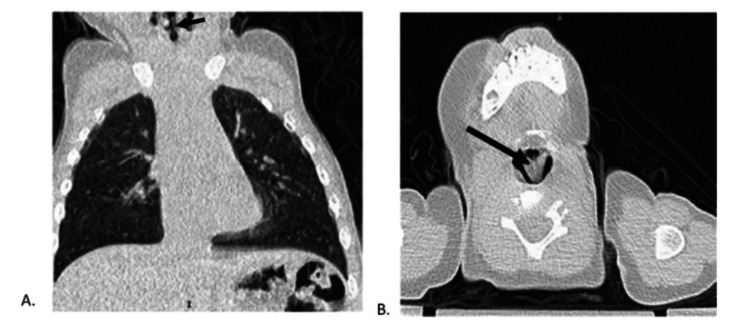
Computed tomography images of the chest A) Chest CT sagittal view demonstrating a thin foreign body in the glottis (arrow), consistent with a thin plastic sequin star B) CT neck axial view further demonstrating a thin plastic foreign body in the glottis.

An 11-month-old male patient with no significant medical history initially presented to the ED via ambulance with vomiting following a witnessed choking event. His parents noted that the patient could have ingested a small plastic object. A physical examination demonstrated inspiratory stridor without wheezing or subcostal retractions. A chest X-ray was obtained, which failed to identify any radiopaque mass or air trapping. To further investigate, due to continued symptoms, a CT of the chest was obtained, which was not reported to show any airway foreign body (Figure 1).

Following administration of dexamethasone and racemic nebulized epinephrine, the patient’s stridor resolved, and he was able to resume oral feeding shortly after. Since he had clinical improvement, he was discharged with instructions to return to the ED if symptoms returned.

The patient’s symptoms returned a few days later. He was treated by his pediatrician with racemic epinephrine and systemic steroids, but ultimately returned to the emergency department eight days after his initial presentation with continued stridor. Physical examination revealed biphasic stridor, aphonia, and elevated blood pressure (106/77 mmHg). ENT was consulted out of concern for upper airway pathology.

**Figure 2:**
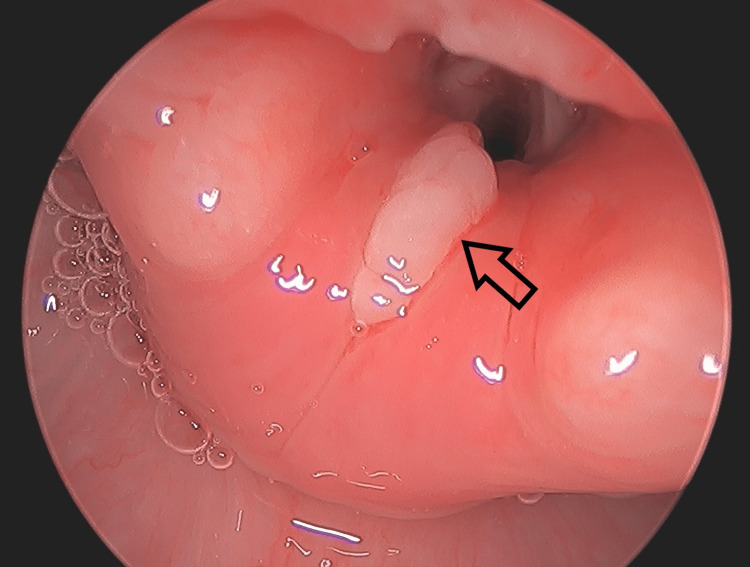
View of the glottis during direct laryngoscopy Granulation tissue (arrow) and edema are seen in the larynx immediately following FB removal.

**Figure 3:**
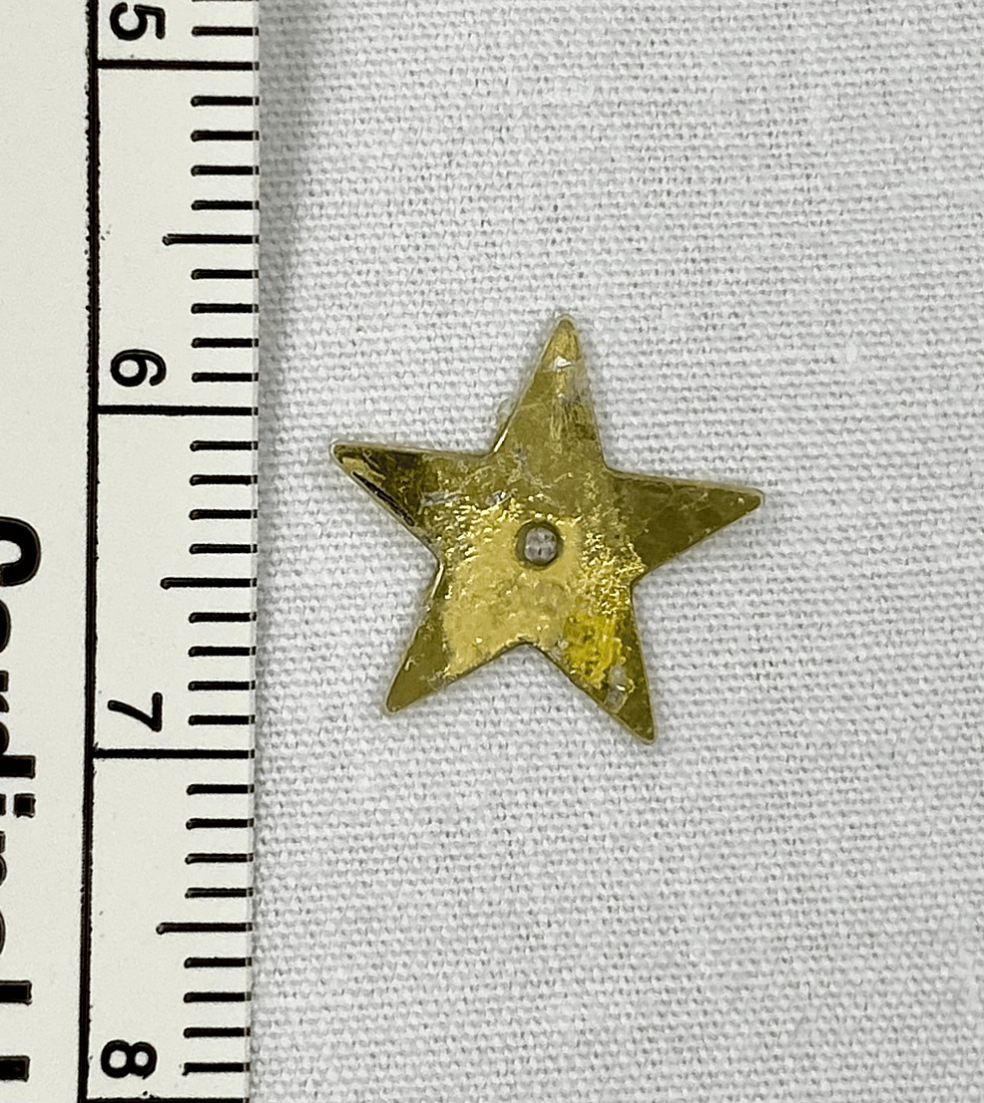
Foreign body retrieved: sequin star approximately 1.2 cm in diameter

Flexible fiberoptic laryngoscopy performed by ENT at bedside revealed a thin gold mass between the vocal folds that did not clear with swallowing or coughing. The previous CT scan was reviewed again by the ENT team. There was noted to be a thin mass at the glottis, which correlated with the abnormal findings seen on laryngoscopy. Suspicion was raised for a retained glottic foreign body, and the patient was brought urgently to the operating room for direct laryngoscopy and bronchoscopy (DLB). A thin star sequin measuring 1.2 cm in diameter was found to be wedged between the vocal cords with granulation tissue overlying the bilateral vocal folds, post-glottis, and subglottis (Figures 2, 3).

The object was removed, and the remainder of the trachea and mainstem bronchi were evaluated without identification of additional foreign bodies. The patient was admitted for observation overnight. Clinical improvement was noted in the morning, and the patient was discharged on a systemic steroid taper and inhaled fluticasone propionate. One month later, a repeat DLB revealed a normal, fully healed larynx.

## Discussion

The larynx is sometimes overlooked for foreign body aspiration due to its low incidence of foreign body impaction and potential nonspecific symptoms. A 2010 literature review examined 12,979 cases of FBA and demonstrated laryngeal or tracheal involvement in only 12% of cases [3]. Another single institution retrospective study identified the larynx as the site of FBA in only nine (8.6%) of the 105 FBA cases [4].

Symptoms of laryngeal FBA, such as stridor, dyspnea, and dysphonia, are non-specific and seen in more common etiologies, such as upper respiratory tract infection, including croup [4,5]. However, unlike other sites, laryngeal FBA may also present with mild or intermittent dysphonia. Thus, appreciation of the subtle changes in a patient’s phonation may suggest laryngeal FBA in the pediatric population [6]. As pediatric patients often rely on their parents to deliver their history, physicians should employ targeted questioning about possible changes in the voice or cry or recent interaction with objects having the potential for aspiration.

Because of the non-specific symptomatology, the diagnosis of FBA often depends on visualizing the object through radiographic imaging. Chest and neck X-rays are particularly effective at detecting the FBA of radiopaque objects. Neck X-rays are more sensitive at assessing laryngeal FBA, as a chest X-ray may not include the glottis within the image margins. However, most cases of FBA in children under the age of three involve the aspiration of radiolucent objects [7]. Lateral decubitus X-rays may assist in the diagnosis of radiolucent foreign bodies due to air trapping from the occlusion of more distal airways. This has a relatively low sensitivity, so suspicion of FBA may remain in the appropriate clinic situation [8].

CT imaging has demonstrated superior sensitivity for the diagnosis of FBA compared to X-rays, especially in the detection of radiolucent objects in the tracheobronchial tree [9]. CT has been shown to reduce the negative bronchoscopy rate [10]. However, false negative imaging readings may contribute to significant delays in the treatment of radiolucent aspirated objects. Studies have shown that if imaging results in a false negative, the identification of radiolucent laryngeal FBA can be delayed by as much as 17.6 days, whereas accurate imaging results can identify laryngeal FBA in just 1.6 days [4]. In our case, the aspirated object, though subtle, was visible on CT imaging, though this finding was not noted in the original reading. Imaging reported to be negative delayed the identification of the LFBA in our patient by eight days.

Fiberoptic flexible laryngoscopy (FFL) facilitates rapid visualization of the airway, yet it inadequately visualizes the subglottis and lacks the capability for forceps-assisted removal of foreign bodies [6]. Direct laryngoscopy and bronchoscopy (DLB) remain the gold standard for diagnosing and managing FBA, as they enable both comprehensive airway visualization, maintenance of ventilation, and the removal of foreign objects under general anesthesia [11]. However, the inherent risks associated with this procedure and anesthesia itself, coupled with resource constraints, render it impractical to perform a DLB on every patient presenting with symptoms consistent with FBA. Consequently, a thorough assessment of the patient’s history and a physical examination, complemented by a targeted diagnostic workup, are imperative before conducting DLB on a stable patient.

Importantly, the absence of abnormalities in imaging studies should not postpone the necessary intervention for DLB. While laryngeal FBA might not initially cause complete airway obstruction, it can lead to significant inflammatory responses that result in persistent respiratory symptoms and potential airway compromise [12]. Therefore, when a patient’s history and clinical presentation, irrespective of imaging outcomes, suggest FBA, immediate intervention via DLB is warranted. This is especially imperative for patients exhibiting signs of respiratory distress or deteriorating respiratory status, as observed in the case under discussion [13].

Missed laryngeal FBA prolongs the disease course and can lead to long-term sequelae, as has been described in previous case reports. Robinson described a case where a nine-year-old, initially misdiagnosed with viral croup, was found to have a small plastic object in the subglottic region. After removing the foreign body, the child required five days of intubation due to worsened respiratory symptoms [14]. Tsuji et al. reported a case where an eight-year-old with a missed laryngeal FBA resulted in severe subglottic stenosis after it was retained for several weeks, thus necessitating a tracheostomy [15]. Others have reported persistent laryngeal bodies. Mallouk et al. reported on a two-year-old female who had mild hoarseness and stridor for five days because of a small plastic mesh material between her vocal cords [16]. Fraccaroli et al. reported a case of a 13-month-old who was misdiagnosed with laryngospasm but then had worsening respiratory symptoms. This led to a DLB being performed, where it was discovered that the patient had aspirated small paper fragments [17]. Rosenthal et al. reported a case of a six-year-old girl with intermittent stridor, difficulty breathing, and hoarseness for nine months due to an embedded laryngeal foreign body [18].

Implementing systems to accurately identify and rapidly treat patients with FBA may improve patient outcomes. For instance, a group in China developed a system involving the implementation of an on-call FBA-dedicated team with an otolaryngologist who would initially review FBA cases. An operating room would be readily available if patients required immediate surgical intervention [19]. Another group developed a predictive statistical model that considered elements of history and physical examination to stratify patients based on the risk of aspiration to help identify those who should undergo DLB. They report a sensitivity of 100% and a specificity of 41% for accurately predicting FBA [20]. While no models exist specifically for laryngeal FBA, the generation of such predictive models with current patient data could assist in stratifying patients with a high likelihood of laryngeal FBA and thus facilitate prompt intervention via DLB.

## Conclusions

Children are especially at risk of foreign body aspiration. The larynx is an uncommon site for lodgment of an airway foreign body that often manifests with non-specific symptomatology, such as dysphonia and stridor. Radiographic imaging may miss thin or radiolucent foreign bodies. This could result in a delay in diagnosis and, thus, an increased risk of long-term sequelae, including airway obstruction. Prompt otolaryngologic evaluation with direct laryngoscopy and bronchoscopy, regardless of imaging results, should be obtained when there is a history of possible aspiration and symptoms of dysphonia or aphonia.
